# Digging deeper into lymphatic vessel formation *in vitro *and *in vivo*

**DOI:** 10.1186/1471-2121-12-29

**Published:** 2011-06-24

**Authors:** Benoit Detry, Françoise Bruyère, Charlotte Erpicum, Jenny Paupert, Françoise Lamaye, Catherine Maillard, Bénédicte Lenoir, Jean-Michel Foidart, Marc Thiry, Agnès Noël

**Affiliations:** 1 Laboratory of Tumor and Development Biology, Groupe Interdisciplinaire de Génoprotéomique appliqué-Recherche (GIGA-Cancer), University of Liège, B-4000 Liège, Belgium; 2 Department of Gynecology, CHU, B-4000 Liège, Belgium; 3 Laboratory of Cell and Tissue Biology, Groupe Interdisciplinaire de Génoprotéomique appliqué-Recherche (GIGA-Neurosciences), University of Liège, B-4000, Liège, Belgium

## Abstract

**Background:**

Abnormal lymphatic vessel formation (lymphangiogenesis) is associated with different pathologies such as cancer, lymphedema, psoriasis and graft rejection. Lymphatic vasculature displays distinctive features than blood vasculature, and mechanisms underlying the formation of new lymphatic vessels during physiological and pathological processes are still poorly documented. Most studies on lymphatic vessel formation are focused on organism development rather than lymphangiogenic events occurring in adults. We have here studied lymphatic vessel formation in two *in vivo *models of pathological lymphangiogenesis (corneal assay and lymphangioma). These data have been confronted to those generated in the recently set up *in vitro *model of lymphatic ring assay. Ultrastructural analyses through Transmission Electron Microscopy (TEM) were performed to investigate tube morphogenesis, an important differentiating process observed during endothelial cell organization into capillary structures.

**Results:**

In both *in vivo *models (lymphangiogenic corneal assay and lymphangioma), migrating lymphatic endothelial cells extended long processes exploring the neighboring environment and organized into cord-like structures. Signs of intense extracellular matrix remodeling were observed extracellularly and inside cytoplasmic vacuoles. The formation of intercellular spaces between endothelial cells led to tube formation. Proliferating lymphatic endothelial cells were detected both at the tips of sprouting capillaries and inside extending sprouts. The different steps of lymphangiogenesis observed *in vivo *are fully recapitulated *in vitro*, in the lymphatic ring assay and include: (1) endothelial cell alignment in cord like structure, (2) intracellular vacuole formation and (3) matrix degradation.

**Conclusions:**

In this study, we are providing evidence for lymphatic vessel formation through tunneling relying on extensive matrix remodeling, migration and alignment of sprouting endothelial cells into tubular structures. In addition, our data emphasize the suitability of the lymphatic ring assay to unravel mechanisms underlying lymphangiogenesis.

## Background

The lymphatic vasculature functions as a tissue drainage system and an immunological control system by collecting extravasated fluid, macromolecules and leukocytes from tissues. The lymphatic system is involved in numerous pathologies such as cancer, lymphedema, inflammation and graft rejection [1-5]. It is also implicated in the dissemination of tumor cells to regional lymph nodes which results in poor prognoses of patients with cancers [6,7]. Reflecting its specialized functions, the lymphatic vasculature displays a distinctive structure. In sharp contrast to blood vessels, the basement membrane of lymphatic vessels is discontinuous or absent. Lymphatic endothelial cells (LEC) display tight junctions and interdigitations, and are connected to the surrounding collagen fibers by anchoring filaments [8-10]. The discovery of specific markers for LECs enabled technical progress in lymphatic vascular biology and greatly promoted lymphatic research [3,4,11].

Although mechanisms leading to new blood vessel formation during physiological and pathological processes are well documented, how migrating LEC organized into new lymphatic vessels has long been a mystery. The prevailing view of their origin from the venous system during embryogenesis is supported by studies performed in mouse and zebrafish [12-16]. LEC could also derive from mesenchymal progenitor cells or lymphangioblasts identified in amphibian and birds through a process referred as lymphvasculogenesis [17,18]. There is an emerging body of work concentrated on attempts to elucidate how to create tubes and generate a complex functional vascular tree [19,20]. Tube morphogenesis is an important morphogenetic process observed during various developmental and pathological events. Regarding epithelial cells, five putative mechanisms have been proposed for tube formation and include: (1) the wrapping of a cell sheet to form a tube; (2) the budding of cells from a pre-existing tube; (3) the cavitation during which the central cells of a solid spheroidal or cylindral mass of cells are eliminated to create a tube; (4) cord hollowing generating a lumen between aggregated cells or (5) cell hollowing creating intracellular luminal spaces inside a single cell, spanning the length of the cell [21]. Progress in understanding the processes of lumen formation (luminogenesis) has benefited from elegant studies in the zebrafish system [16,22] and *in vitro *models of tubulogenesis [23,24] and of sprouting angiogenesis in 3D extracellular matrix (ECM) environments [25,26]. For blood vessel formation, it is now widely accepted that blood endothelial cells (BEC) at the tip of the bud (named tip cells) invade the matrix and create a space that can be occupied by a cord of cells without apparent lumen. Behind the tip cell, the so-called "stalk cells" composing the stalk of the sprouting capillary are proliferating and contribute to stalk elongation, as well as to basement membrane deposition [27]. BEC organization along the matrix space generated by migrating cells initiates an extracellular luminal area resulting in the transformation of cord into a tube [19,20]. Cell hollowing or intracellular vacuolization is an additional mechanism by which individual cells generate vesicles that can interconnect with adjacent cells leading to lumen size increase. In sharp contrast to those major advances made in the field of angiogenesis, little information is available on how LEC migrate and organize into lymphatic vessels during lymphangiogenesis. Although lymphatic vessels are enclosed in a matrix structure mainly composed of collagens, the extent of ECM remodeling in lymphangiogenesis is unclear. A major challenge is the difficulty of establishing appropriate *in vivo *models and culture systems to enable the dissection of this complex biological process. Recently, several *in vivo *and *in vitro *models of lymphangiogenesis have been developed and are useful for exploring the cellular and molecular mechanisms of lymphangiogenesis [13,28-33].

In the present study, ultrastructural features of neoformed lymphatic vessels have been investigated in two *in vivo *models and one *in vitro *3D culture system: (1) the corneal lymphangiogenic assay induced by thermal cauterization of the mouse cornea [34]; (2) the lymphangioma model consisting in lymphatic cell hyperplasia induced by intra-peritoneal injection of incomplete Freund's adjuvant [35-37] and (3) the lymphatic ring assay which bridges the gap between *in vitro *and *in vivo *systems [38,39]. We provide innovative morphological data, at the ultrastructural level, demonstrating the pronounced ECM remodeling and intracellular vacuolization during the migration, alignment and organization of channels of sprouting lymphatic cells *in vivo*. Through Transmission Electron Microscopy (TEM), we show that collagen degradation takes place as an important step for vessel neoformation during lymphangiogenesis.

## Results

### Induction of lymphangiogenesis *in vivo*

**Figure 1:**
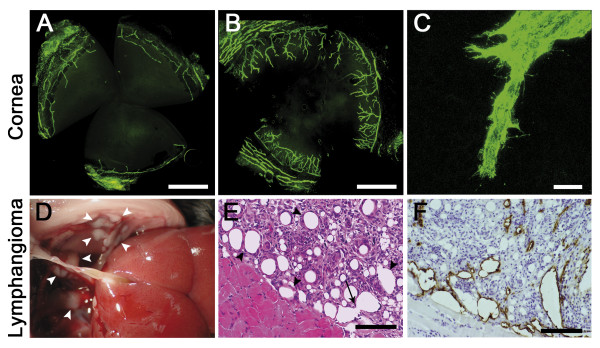
**Lymphangiogenesis *in vivo***. *(****A-C****): Corneal lymphangiogenesis induced by thermal cauterization*. Corneal flatmounts are labeled in green with an anti-LYVE-1 antibody and observed on fluorescent (**A, B**) or confocal (**C**) microscope. (**A**): In normal condition, cornea is avascular and the limbal vascular arcade is positive for LYVE-1 staining. (**B**, **C**): Seven days after an inflammatory stimulus, lymphatic vessels outgrow from the limbus towards the central cornea. (**C**): Migrating LEC display filopodia-like structures. *(****D-F****): Mouse lymphangioma induced by intraperitoneal injection of incomplete Freund's adjuvant*. (**D**): Lymphangiomas appear as white masses at the surface of the diaphragm (white arrowheads). (**E**): Hematoxylin-eosin staining of a histological section of the diaphragm (black arrowheads: lymph vessels) (**F**): Lymphatic vessels are evidenced by LYVE-1 immunostaining. The arrow delineated the process of fusion leading to increased lumen size. Scale bars in (**A**, **B**): 1 mm, in (**C**): 20 μm, and in (**E**, **F**): 100 μm.

To investigate the mechanism leading to lymphatic vessel neoformation, we used three distinct models of lymphangiogenesis in a collagen rich environment. The mouse model of thermal cauterization-induced corneal lymphangiogenesis mimicks lymphangiogenesis occurring upon inflammatory conditions such as keratitis (from viral or bacterial origin), chemical burns and graft rejection [1,34,40,41]. Although the cornea is an avascular tissue, upon inflammatory "insult" such as thermal cauterization, LYVE-1 positive lymphatic vessels arose perpendicularly from the limbal vascular arcade (Figure 1A, B). Upon confocal microscopy, LEC at the end of branching vessels displayed numerous filopodia-like extensions reflecting their migrative feature (Figure 1C). In the second *in vivo *model used, LEC hyperplasia was induced by intra-peritoneal incomplete Freund's adjuvant injection. White masses of lymphangioma appearing at the surface of the diaphragm were collected one month after the first injection (Figure 1D). Lymphatic vessels were visible upon hematoxylin-eosin staining (Figure 1E) and were LYVE-1 positive (Figure 1F). Various levels of cell fusion are observable leading to the progressive increase of vessel-like lumen size.

### Ultrastructural features of lymphangiogenesis *in vivo*

**Figure 2:**
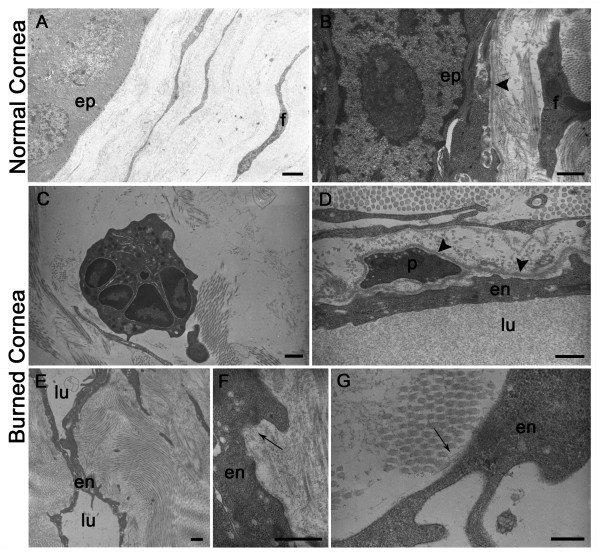
**Electron microscopy of normal or burned mouse cornea**. (**A, B**): Normal cornea reveals epithelial cells (ep) apposed on a regular basement membrane (arrowhead in B). Flattened fibroblastic cells (f) are surrounded by collagen fibrils in 2 perpendicular orientations. (**C**-**G**): burned cornea. (**C**): A neutrophil is seen in a remodeled collagen matrix. (**D**): A blood capillary lined by an endothelial cell (en) is surrounded by a continuous basal lamina (arrowhead) in which is incorporated a pericyte (p). (**E**): A lymphatic capillary is shown with a narrow irregular lumen (lu). (**F, G**): LEC (en) are associated with bundles of thin anchoring filaments into collagen fibrils (arrows). en = endothelial cell; lu = lumen. Scale bars in (**A**, **E**): 2 μm, in (**B-D**): 1 μm, and in (**F**, **G**): 0.5 μm.

We first examined by TEM the normal cornea that is composed of a multi-layered cellular epithelium and a connective tissue stroma which makes up the bulk of the cornea (Figure 2A). Basal epithelial cells were apposed on a regular basement membrane (Figure 2B). The stroma was formed by several lamellas of parallel collagenous bundles which crossed at an angle to each others. The collagen fibrils within each lamella were parallel to each other and ran the full length of the cornea. Stromal fibroblasts appeared as elongated flattened cells interspaced with collagen in the cornea. These cells were characterized by a very thin cytoplasm devoid of vacuoles (Figure 2A, B). As expected, the normal cornea was devoid of any blood or lymphatic vessels. After thermal cauterization, inflammatory cells such as neutrophils were observed in a remodeled collagen matrix (Figure 2C). Sprouting blood and lymphatic endothelial cells were morphologically identified in accordance with previous reports [42,43]. Neo-formed blood vessels often contained white or red blood cells and were characterized by the presence of a continuous basement membrane that frequently surrounded a pericyte (Figure 2D). In contrast to blood vessels, lymphatic vessels displayed an irregular and narrow lumen (Figure 2E). LEC of neo-formed lymphatic capillaries were joined by interdigitations and were distinguishable by their intimate association with collagen fibrils through anchoring filaments and the absence of a continuous basement membrane (Figure 2F, G).

**Figure 3:**
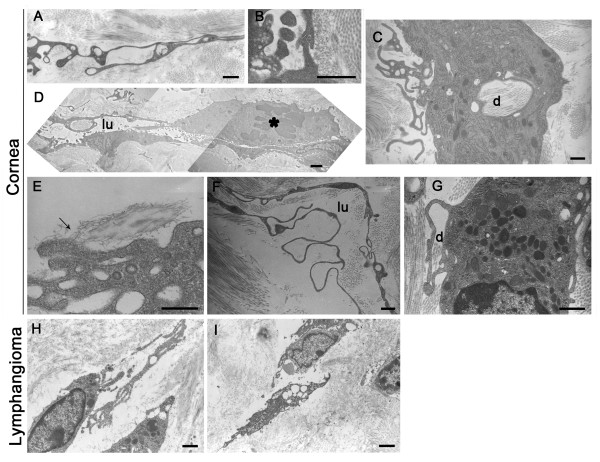
**Electron microscopy pictures of lymphangiogenesis *in vivo***. Lymphangiogenesis was observed after thermal cauterization of the cornea (**A-G**) and in lymphangioma (**H**, **I**). (**A**): Lymphatic endothelial cells (LEC) form long processes containing vesicles and delimitating extracellular spaces devoid of matrix or with reminiscent matrix fragments. Note the presence of intracellular vesicles in endothelial processes. (**B**): Endothelial cells are joined by interdigitations. (**C**): Intracellular vesicle contains matrix fragments (d). (**D**): Aligned endothelial cells form a tubular structure that delimits a narrow lumen (lu). The luminal surface of endothelial cells is ruffled with small cell processes. A mitotic endothelial cell is visible (*). (**E**): LEC are anchored to the matrix through anchoring filaments (arrow). (**F**): Tubular structures containing a lumen (lu) are lined by long cytoplasmic extensions of LEC. (**G**): Connection of two cell extensions delineates an extracellular space containing degradation products of the matrix that are reminiscent of collagen fibrils. (**H, I**): LEC are aligned and surrounded by matrix-free extracellular spaces. Note the presence of coalescent vacuoles. Scale bars in (**A-C**; **F-H**): 1 μm, in (**D, I**): 2 μm, and in (**E**): 0.5 μm.

During the process of lymphatic vessel formation, migrating LEC extended long processes (Figure 3A, C) and aligned to organize into cord-like structures (Figure 3D). They progressively interconnected by interdigitations (Figure 3B) and adhered to the collagen matrix through anchoring filaments (Figure 3E). The presence of mitotic figures reflects the proliferating feature of these LEC forming neo-vessels (Figure 3D). Gaps were often observed between neighboring cells. Extracellular spaces formed also thin tubular structures incompletely lined with elongated cells (Figure 3A, D, F). The continuity of the endothelial lining was provided by the cytoplasmic processes of LEC that formed interdigitating, overlapping and end-to-end junctions, finally delimitating a lumen and forming a so-called prelymphatic vessel (Figure 3D, F). During these events, noticeable signs of ECM remodeling and intracellular collagen degradation were detected, including the presence of a large amount of lysosomes (Figure 3C, G). The TEM analysis of lymphangioma largely confirmed the observations made on the cornea and provided evidence for the establishment of intercellular spaces leading to tubular structures (Figure 3H, I). LEC alignment into cords with a thin and irregular lumen was also noticed (Figure 3I). Reminiscent matrix fragments resulting from matrix degradation were again detected in interendothelial gaps and in the lumen of neo-formed vessels (Figure 3H, I).

**Figure 4:**
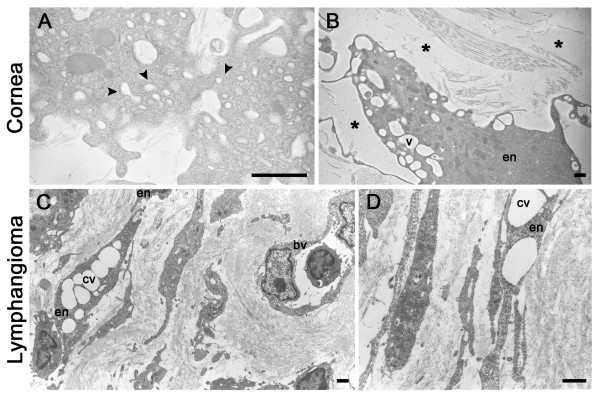
**Vacuolization and lumen formation during lymphangiogenesis *in vivo***. Lymphangiogenesis was observed after thermal cauterization of the cornea (**A, B**) and in lymphangioma (**C, D**). (**A**): Prominent pinocytic activity (arrowhead) is visible along the plasma membrane and at cell junction. (**B**) Endothelial cells (en) containing intracellular vesicles are aligned and surrounded by matrix-free extracellular spaces (*). (**C**): Aligned elongated endothelial cells surrounded by extracellular spaces. Note the coalescence of intracellular vesicles (cv) and the presence of a blood vessel (bv) containing a white cell. (**D**) Vesicle coalescence (cv) into an intracellular luminal space is visible through a process similar to that depicted in **B**. bv = blood vessel; cv = coalescent vesicle; en: endothelial cell. Scale bars: 1 μm.

Migrating cells displayed numerous intracellular vacuoles of variable size, including in their cytoplasmic extension (Figure 3A, H and Figure 4A, B). The intracellular vacuoles fused to form a large intracellular luminal cavity (Figure 3I, Figure 4B, C, D). In addition, the establishment of intercellular spaces between LEC cords or LEC processes and the connection to and fusion with each other led to lumen formation (Figure 3D). Similar observations were made in both *in vivo *models.

### The lymphatic ring assay reproduces *in vitro *the lymphangiogenic process

**Figure 5:**
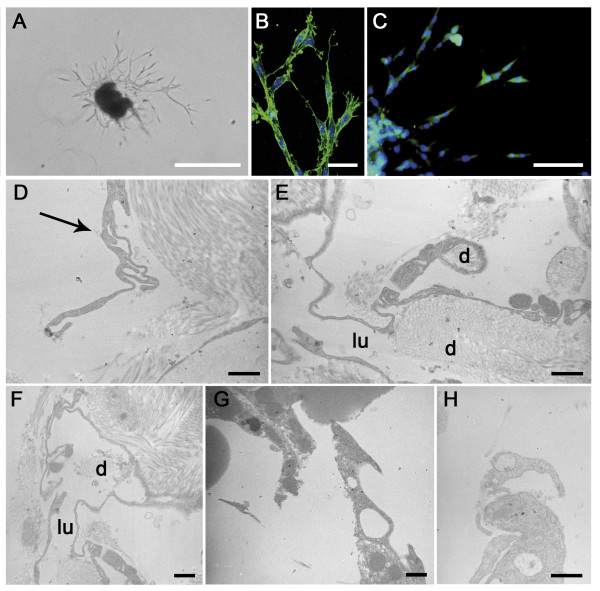
**Lymphatic ring assay**. (**A**): Visualization by optical microscopy of LEC spreading from mouse thoracic duct fragment embedded for 11 days in a 3D-type I collagen gel. (**B**): Outgrowing LEC display filopodia-like structures visible upon phalloidin staining under confocal microscopy. (**C**) LEC are labeled in green with an anti-LYVE-1 antibody and observed under a fluorescent microscope. Nuclei are counterstained in blue (Dapi). (**D-H**) Electron microscopy micrographs of 3D-cultures of lymphatic thoracic duct rings. (**D**): Long extended processes of LEC. Note the invagination process (arrow). (**E, F**): Establishment of an irregular lumen in tubular structures lined by long thin endothelial cell processes. (**G, H**): Sprouting endothelial cells display processes and intracellular vesicles. d = matrix degradation. d = matrix degradation; lu = lumen. Scale bars in (**A**): 500 μm, in (**B**): 40 μm, in (**C**): 100 μm, and in (**D-H**): 1 μm.

**Figure 6:**
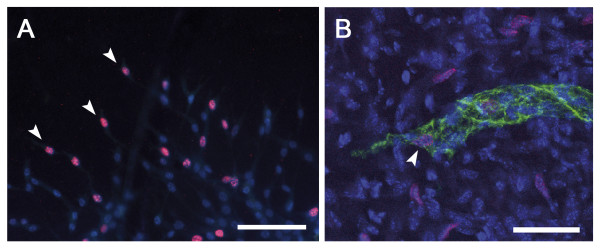
**LEC proliferation during lymphangiogenesis**. Cell proliferation was visualised by immunostaining following BrdU incorporation (red) *in vitro*, in the lymphatic ring assay (A) and *in vivo*, in whole mounted burned cornea (B). Proliferating LEC at the tip of capillary bud (arrowhead) are detected both *in vitro *(A) and *in vivo *(B). LEC are labeled in green with an anti-LYVE-1 antibody (B). Nuclei are counterstained in blue with Dapi (A) or TO-PRO3 (B). The whole mounted samples are observed under fluorescent (A) or confocal (B) microscope. Scale bars in (**A**): 100 μm and in (**B**): 40 μm.

To validate the *in vivo *observations, we then used the lymphatic ring assay which bridges the gap between *in vivo *and *in vitro *systems and recapitulates, in a collagen environment, the different steps of cell sprouting from a pre-existing lymphatic vessel [38]. In these 3D lymphatic ring cultures (Figure 5A), LYVE-1 positive endothelial sprouts (Figure 5C) first appeared after 5 days of culture under a 5% O_2 _atmosphere and reached a maximal outgrowth after 11 days. The observations upon confocal microscopy revealed that neovessel tips were made of migrating cells which extended filopodia-like processes probing the surrounding matrix (Figure 5B). Recent findings in the field of angiogenesis led to the identification of specialized endothelial cells including the tip cells that are non proliferating cells probing the environment at the extremities of endothelial bud; and stalk cells that proliferate and elongate the stalk of the sprout. We thus explored the proliferation rate of migrating cells in sprouting capillaries through BrdU incorporation. Both migrating cells at the tip of sprouting capillaries and cells inside the extending sprout incorporated BrdU (Figure 6A). The percentage of proliferating cells was 40 ± 14% at the extremities and 21 ± 5% inside the forming buds. The proliferative feature of LEC at the tips of extending sprouts was confirmed *in vivo *in the corneal assay (Figure 6B).

**Figure 7:**
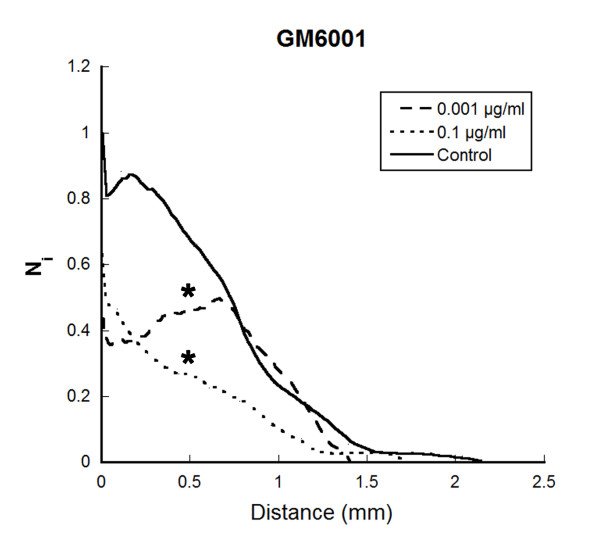
**Modulation of lymphatic vessel outgrowth by protease inhibitor**. Lymphatic rings cultured in the presence of serum were treated with a broad spectrum MMP inhibitor (GM6001) at 0.1 μg/ml and 0.001 μg/ml. Quantification of LEC sprouting was measured by determining the number of intersections between capillaries and a grid obtained by the dilatation of the ring boundary, as previously described [38]. The number of intersections (Ni) is plotted as a function of the distance to the ring. * = P < 0.05.

As the outgrowth expanded, vessels developed a visible lumen as previously reported [38]. The electron microscopic findings supported the data generated *in vivo*. Indeed, the sprouting LEC showed again intracellular vesicles in their cytoplasm, as well as in the numerous processes that they extended (Figure 5D, G, H). Degradation products of collagen were also visible in intracellular vesicles, in intercellular spaces of tubular structures and in extracellular spaces delimitated by pseudopode-like extensions of migrating cells (Figure 5E, F). The putative implication of proteases of the matrix metalloprotease (MMP) family was assessed in this model, by using synthetic MMP inhibitor (Figure 7). Lymphatic vessel outgrowth was inhibited in a dose-dependent manner by the broad-spectrum inhibitor GM6001 (Figure 7).

## Discussion

**Figure 8:**
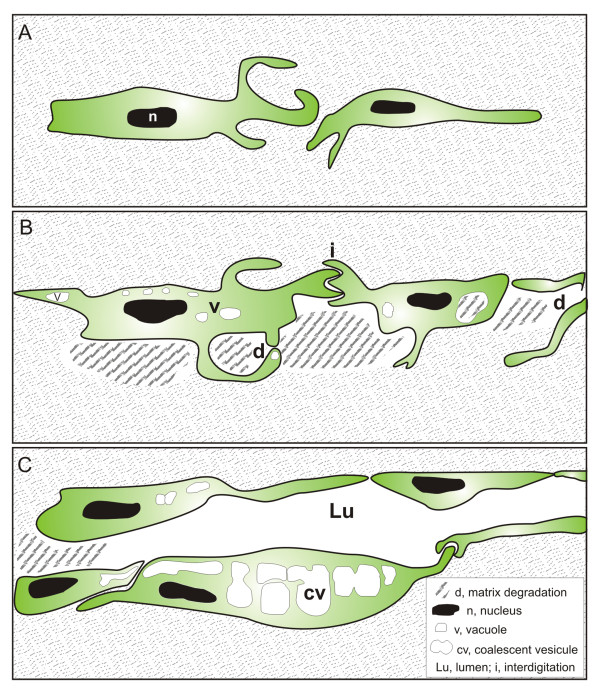
**Tunneling model of lymphatic vessel formation**. The model is based on ultrastructural observations performed in *in vitro *and *in vivo *models of lymphangiogenesis. (**A**): LEC alignment. Elongated LEC migrate and extend long cytoplasmic protrusions. (**B**): Vacuolization and matrix degradation. The continuity of LEC lining is mediated by interdigitations (i). Vesicle invaginations lead to the formation of intracellular vacuoles (v) in the cytoplasm and in protrusions. Matrix degradation (d) occurs intracellularly and extracellularly generating space between cells. (**C**): Luminogenesis. The lumen (lu) is formed *de novo *in the intercellular space. The intracellular vacuoles coalesce (cv) and likely fuse with the cytoplasmic membrane to increase the lumen.

Recent studies on lymphatic vessel formation have mainly focused their interest on organism development. On the contrary, much less is known about the process of lymphangiogenesis occurring in pathological conditions. This study sought to define the ultrastructural features of neo-formed lymphatic vessels and exploited the attributes of two established models of inflammation accompanied by a robust lymphangiogenesis [35,36,40], and the advantage of the recently set up model of lymphatic ring assay which recapitulates all steps of sprouting lymphangiogenesis [38]. Here, we propose a model of lymphatic vessel formation through tunneling (Figure 8). This concept is supported by similar TEM observations generated in three distinct models demonstrating that the formation of lymphatic neo-vessels relies on the alignment of LEC which drive a tunnel through extracellular matrix. During lymphangiogenesis, cords of cells create an extracellular space by the degradation of collagen fibrils occurring extracellularly and intracellularly. Sprouting LEC are characterized by (1) the extension of long thin vacuolized processes which probe the extracellular environment (Figure 8A), connect with adjacent cells resulting in the formation of cord-like structures and pre-lymphatic vessels consisting in thin tubular structures lined with elongated LEC (Figure 8B); (2) an intense intracellular vacuolization associated with vesicle coalescence leading to an intracellular luminal space (Figure 8B, C); (3) a matrix remodeling generating space between cells promoting cell migration and contributing to lumen formation (Figure 8B, C). Furthermore, the present study underlines the strength of the *in vitro *lymphatic ring assay which recapitulates the processes observed *in vivo *in pathological conditions.

Emerging descriptions of cellular and molecular events of tubulogenesis occuring during blood vessel formation have converged on three mechanisms underlying angiogenesis: budding (or sprouting), cord hollowing and cell hollowing [19,44,45]. Progress in understanding such angiogenic tube morphogenesis has benefited from 3D culture systems. The present study represents the first ultrastructural description of capillary formation during pathological lymphangiogenesis. In line with the previous descriptions of the angiogenic process, we observed intracellular and extracellular hollowing events. A common feature of the three lymphangiogenic processes studied here is the migration of cells creating spaces that can be occupied by a cord of very thin and elongated cells delimitating a luminal space. In the present study, the involvement of cell proliferation has also been evidenced during cord formation. Cell hollowing or intracellular vacuolization is a mechanism by which individual cells generate vesicles that can enable the cells to interconnect with neighboring cells to form multicellular lumens and tubes [46-48]. Cell vacuolization is a common feature of migrating cells in the three models presented here. Vesicles of various sizes were seen to progressively enlarge and fuse to each other to, in turn, form a large intracellular luminal vesicle. By analogy with the angiogenic process, this space likely fuses with vesicle of adjacent cells to form the lumen of a pre-lymphatic vessel. This concept is supported by the process of cell fusion leading to increased lumen size clearly seen in the lymphangioma both at ultrastructural and histological levels (Figures 1 and 3). The intracellular vacuolization mechanism was initially associated with the morphogenesis of single endothelial cells which had no contact with adjacent cells occurring during the process of vasculogenesis [44,49,50]. The intracellular vacuolization has been extensively studied in tubulogenesis assay on 3D matrix leading to the identification of key molecular regulators such as matrix metalloproteinases and small GTPase [46,47,51]. In this context, the zebrafish system was suitable to demonstrate the importance of such process in an *in vivo *context during developmental conditions [48]. The present ultrastructural investigation provides the first evidence of intracellular vacuolization *in vivo *during lymphangiogenesis. Further investigations are required to give new molecular insights on how this process contributes to lumen formation in lymphatic capillaries. Despite further attempts, we have been unable to set up a real-time visualization of living cells with confocal or two photons microscopes in the lymphatic ring assay.

An exciting advance in the field of angiogenesis came from the finding that several types of specialized endothelial cells (tip cells and stalk cells) are involved in the building of functional blood capillaries. It has been described that lymphatic tip cells expressed more vascular endothelial growth factor receptor-3 (VEGFR3) and neuropilin-2 [52] but the transposition of the new concept of tip/stalk cells from angiogenic sprouts to lymphangiogenic sprouts in terms of cell proliferation is still premature. In order to shed some light on this issue, we have analyzed the proliferation rate of migrating cells in sprouting capillaries, both *in vivo *in the corneal assay, and *in vitro *in the lymphatic ring assay. Proliferation assessed by BrdU incorporation was observed both in extending capillaries and at their extremities. In the aortic ring that mimicks the angiogenic process, a quantitative analysis of proliferating cells revealed that none of the tip cells had incorporated BrdU, while 12 ± 5% of the stalk cells were BrdU positive (data not shown). These data suggest that the concept of tip cells defined as non proliferating cells probing the environment can not be extended to the process of lymphangiogenesis and emphasizes differences between the cellular mechanisms underlying lymphangiogenesis and angiogenesis.

Of great interest is our finding that LEC create *in vivo*, physical spaces within the surrounding collagen rich environment. This is associated with an extensive extracellular matrix remodeling both evidenced extracellular and intracellularly. Long processes extended by LEC were seen to roll up to enclose matrix fragments and create extracellular spaces. The contribution of MMPs in this remodeling process is supported by the inhibition of LEC sprouting achieved by using a synthetic MMP inhibitor. Such observation is in line with our recent identification of the metalloproteinase-2 (MMP2) which displays collagenolytic activity [53] as a key regulator of lymphangiogenesis [38]. Indeed, the embedding of lymphatic duct fragments issued from MMP2-deficient mice led to impaired LEC sprouting and lymphangiogenic response [38]. The involvement of MMP-driven proteolysis in the lymphangiogenic process is further supported by our previous work using broad spectrum MMP inhibitors in the corneal assay [34]. It is worth noting that intracellular vacuolization and extracellular remodeling are not two exclusive mechanisms (Figure 8). They have been both evidenced in the three distinct *in vitro *and *in vivo *models used here and thus likely operate concomitantly during lymphangiogenesis. Altogether, our data emphasize the interest of the lymphatic ring assay to unravel the cellular and molecular mechanisms of lymphangiogenesis. It appropriately recapitulates *in vitro *the different steps of lymphangiogenesis observed in animal models such as corneal lymphangiogenesis and lymphangioma. The novel emerging panel of *in vitro *and *in vivo *models of lymphangiogenesis [13,30] are suitable to investigate the biology of lymphangiogenesis. This is mandatory for the understanding of several pathological processes such as lymphedema, graft rejection and metastatic dissemination through the lymphatic way.

## Conclusions

The present study provides new insights into lymphangiogenic tube formation. It also highlights the suitability of the lymphatic ring assay to investigate lymphangiogenesis associated with different pathological processes.

## Methods

### Animals

C57BL/6 mice of either sex, 6 to 8 weeks old, were purchased from Janvier (Saint Berthevin, France). All experimental procedures were performed in accordance to the guidelines of the University of Liège regarding the care and use of laboratory animals.

### Corneal assay

Corneal lymphangiogenesis was induced by thermal cauterization of the anesthetized central cornea (Unicaïne 0.4%, Thea Pharma, Wetteren, Belgium) by using an ophthalmic cautery (OPTEMP II V, Alcon Surgial, Fort Worth, USA) on mice anesthetised by intraperitoneal injection of ketamine hydrochloride and xylazine (100 mg/kg and 10 mg/kg, respectively) [34]. Seven days later, mice were sacrificed, eyes were removed and corneas were isolated. In some assays, mice were intraperitoneally injected with Bromodeoxyuridine (BrdU, 200 ul) (Merck, Overijse, Belgium), 2 h before sacrifice. Corneas were stained as whole mount after 1 h fixation in ethanol 70%, at room temperature. Whole mounts were blocked in 3% BSA-3% Gloria milk for 1 h and incubated overnight with polyclonal goat anti-mouse LYVE-1 (1/200, R&D System, Abingdon, UK) or mouse anti-BrdU antibody (1/250, Becton Dickinson, Erembodegem, Belgium). After four washes with PBS, corneas were incubated, for 2 h, with Alexa-Fluor 488 coupled rabbit anti-goat antibody (1/200, Molecular Probes, Merelbeke, Belgium) or TRITC coupled rabbit anti-mouse antibody (1/40, Dako, Glostrup, Denmark). Corneas were flatmounted on a microscope slide with Vectashield mounting medium (Vector Laboratories, Burlingame, CA) and visualized by using a fluorescent microscope (AH3-RFCA, Olympus, Hamburg, Germany) or a LeicaTCS SP2 inverted confocal microscope (Leica Microsystems, Wetzl, Germany).

### Lymphangioma

Lymphangioma or lymphatic endothelial hyperplasia was induced by two intraperitoneal injections of incomplete Freund's adjuvant with a 15-day interval, as described [35,36]. For ethical purposes, buprenorphine injections (0.05 mg/kg) were administered 1 h before and after adjuvant injections, as well as every 12 h during the first 5 days post-injection. After 4 weeks, mice were killed and diaphragms were harvested, fixed in 10% formalin and paraffin embedded. Sections of 4-6 μm thickness were cut and either hematoxylin-eosin stained or immunolabeled by using an anti-LYVE-1 antibody as previously described [35].

### Lymphatic ring assay (LRA)

Thoracic ducts used for lymphatic ring cultures were collected from male and female C57BL/6 mice. Three-dimensional lymphatic ring cultures were carried out as previously described [38,39]. Ring-shaped explants embedded in rat tail interstitial collagen-I gel cultured in MCDB131 (GIBCO, Merelbeke, Belgium) medium supplemented with either 4% Ultroser (BioSepra, Cergy Saint Cristophe, France) or 10% Fetal Bovine Serum (FBS). Cultures were kept at 37°C in a humidified incubator (HERAcell 150, Heraeus, Hanau, Denmark) under hypoxic conditions (5% O_2_, 5% CO_2 _and 90% N_2_) for 11 days. In some assays, MMP inhibitor (GM6001) was added at indicated doses, in the culture medium at the beginning of the experiment. To assess cell proliferation, rings were incubated with BrdU for 3 hours before fixation and immunostaining. For the immunochemistry of whole mounted rings, cultures were fixed in ethanol 70% for staining with rabbit Lyve-1 antibody (1/600, a kind gift from Kari Alitalo, Finland) or with anti-BrdU antibody (1/250, Becton Dickinson, Erembodegem, Belgium). After washes, lymphatic rings were incubated with FITC coupled swine anti-rabbit antibody (1/40) or FITC conjugated rabbit anti-mouse antibody (1/40, both from Dako, Glostrup, Denmark). For FITC coupled phalloidin labeling (Sigma-Aldrich, Schnelldorf, Germany) rings were fixed in paraformaldehyde (4%) [39]. Nuclei were evidenced by TO-PRO3 and Vectashield Dapi (Molecular Probe, Merelbeke, Belgium). Lymphatic capillaries were visualized under a Leica TCS SP2 confocal microscope (Leica Microsystems, Wetzl, Germany) or a fluorescent microscope (AH3-RFCA, Olympus, Hamburg, Germany). Quantification of LEC sprouting was performed by computerized-assisted method as previously described [38,39].

### Transmission Electron Microscopy (TEM)

Samples (lymphatic ring gels, lymphangioma or cornea) were washed in Sörensen's buffer and fixed for 1 h at 4°C with 2.5% glutaraldehyde in a Sörensen 0.1 M phosphate buffer (pH 7.4) and post-fixed for 30 min with 1% osmium tetroxide. After dehydration in graded ethanol, samples were embedded in Epon. Ultrathin sections obtained with a Reichert Ultracut S ultramicrotome were contrasted with uranyl acetate and lead citrate. Observations were made with a Jeol 100 CX II transmission electron microscope at 60 kV.

## Competing interests

The authors declare that they have no competing interests.

## Authors' contributions

BD carried out the corneal assay. FB was responsible for the lymphangioma and lymphatic ring assays. CE, JP, CM, BL contributed to immunostainings and data analysis. FL performed sample preparation for ultrastructural observations. JMF contributed to study supervision. MT was responsible for ultrastructural observations and critically evaluated the data. FB, BD and AN performed TEM observations with MT. AN designed, coordinated the study and wrote the manuscript. All authors contributed to data analysis, manuscript preparation and approved the final manuscript.
